# Experimental Investigation on a Cable Structure Equipped with an Electrodynamic Damper and Its Monitoring Strategy through Energy Harvesting

**DOI:** 10.3390/s19112631

**Published:** 2019-06-10

**Authors:** Seungkyung Kye, Hyung-Jo Jung, Ho-Yeon Jung

**Affiliations:** 1 Department of Civil and Environmental Engineering, Korea Advanced Institute of Science and Technology, 291 Daehak-ro, Yuseong-gu, Daejeon 34140, Korea; skkye@kaist.ac.kr; 2 Advanced Technology Department, Hyundai Construction Equipment, 17-10, Mabuk-ro 240 beon-gil, Giheung-gu, Yongin-si, Gyeonggi-do 16891, Korea

**Keywords:** cable, vibration control, electrodynamic damper, energy harvesting, monitoring

## Abstract

The vibration of cables in a cable-supported bridge affects its serviceability and safety. Therefore, cable dampers are essential for vibration control, monitoring, and the further maintenance of such bridges. In this study, the vibration control performance of an electrodynamic damper applied to a cable used in a footbridge was experimentally verified considering the major design variables of the damper. The damping performance was analyzed by varying the damping ratio according to the excitation condition and external circuit resistance. The acceleration and displacement at each measurement point and the frequency-domain response decreased. Considering the dominant response conditions of the cables in the bridge, an effective additional attenuation was observed. In addition, the harvesting power considering the measurement frequency and power loss was sufficient to operate a wireless measuring sensor by examining the energy harvesting performance from the cable measurement data of an actual bridge in service. Finally, a stepwise operation strategy for a cable vibration monitoring system was suggested and examined by analyzing the meteorological observation data and the power output according to the wind environment. The results demonstrate the feasibility of using an electrodynamic damper to build an integrated monitoring system capable of simultaneous cable vibration reduction and energy harvesting.

## 1. Introduction

The construction of massive civil structures, such as long-span bridges, is on the rise. According to the Korea Institute of Construction Technology, the size of the global long-span bridges market has increased sharply since 2011, and there has been a steady increase since then [1]. Most long-span bridges are cable-supported bridges, in which the main tower and the bridge deck are connected by cables. Thus, the cable is designed to serve as the main structural member [2]. The construction of cable-supported bridges is steadily increasing, because they can reduce construction volume and make the bridge lighter, thereby enhancing scenic beauty while also extending span ranges [3].

Cables, which are the main members of cable-supported bridges, are exposed to continuous loads such as vehicle and wind loads. They are under a risk of being instantly exposed to large loads such as those during earthquakes and typhoons. In the case of turbulent flow, these loads may be dynamically combined with the ambient load, posing a serious risk to bridges [4]. Therefore, continuous monitoring and maintenance of cable vibration is necessary to manage cable bridges effectively and efficiently while maintaining their serviceability and safety.

Various types of vibration control devices have been studied for effective cable vibration control [5,6]. Vibrations that can cause cable serviceability and safety problems, such as during earthquakes and typhoons, are powerful; however, they rarely occur. Therefore, introducing a vibration control device to counter the extremely rare overload is inefficient in terms of cost. In recent years, there has been a growing interest in efficient vibration control and maintenance measures for structures, as the dampers used in the past, such as hydraulic viscous dampers and friction dampers, have a high maintenance cost [7]. Recently, studies on electrodynamic dampers, which are regenerative damping devices, have been actively pursued as effective vibration reduction and maintenance measures for structures.

An eddy current-based damper has been implemented based on the interaction of magnetic forces against magnetic flux changes [8,9]. The dissipation of eddy currents due to the internal resistance of the magnetic material induces a damping effect as the energy of the system is removed. The damping devices based on eddy currents are used in vehicle suspensions [10,11,12,13], vehicle brakes [14,15,16,17], and attenuators of high-rise structures [18,19,20,21]. Further research is underway to apply them to various fields. However, although an eddy current damper has a higher damping force than a coil-based electrodynamic damper, the former can only be used for a passive-type control, because the internal resistance of the magnetic body cannot be controlled [22,23].

On the other hand, electrodynamic dampers can be used for passive, semi-active, or active control, making them more advantageous than eddy current dampers. An electrodynamic damper is a vibration control device based on the electromagnetic induction law, similar to an eddy current damper. It converts the vibration energy of a structure into electric energy. The damping force generated in the conversion process can be used as the vibration control force for the structure, and the electric energy obtained through the electromagnetic induction can be used for monitoring the structure. Recently, research on electromagnetic devices using inerter has been reported. Inerter can be applied to tuned mass damper with power conversion devices such as rack-and-pinion or flywheel, or tuned viscous mass to perform attenuation and energy harvesting [24,25,26]. Although studies on applying electrodynamic dampers to civil engineering structures are actively conducted, most of them are based on numerical analyses. In the case of experimental studies, the verification was performed on a reduced model [27,28]. In addition, it is necessary to verify the energy harvesting performance of electrodynamic dampers under actual wind loads.

The objective of this research was to construct an efficient vibration reduction and monitoring system using an electrodynamic damper. The core parameters of the system are damping and energy harvesting performance. In this study, the electrodynamic damper was fabricated based on the consideration of the major design parameters of the damper. The response characteristics of the damper mounted on the footbridge cable were investigated experimentally. Then, the energy harvesting circuit was fabricated and connected to the electrodynamic damper, and the power harvesting performance based on the measurement data of the actual cable was examined. Finally, based on the energy harvesting performance of the electrodynamic damper, an operational strategy for the construction of the monitoring system was presented and its feasibility under the real wind vibration environment was examined.

## 2. Design Variables of Electrodynamic Damper and Energy Harvesting Circuit Configuration

**Figure 1:**
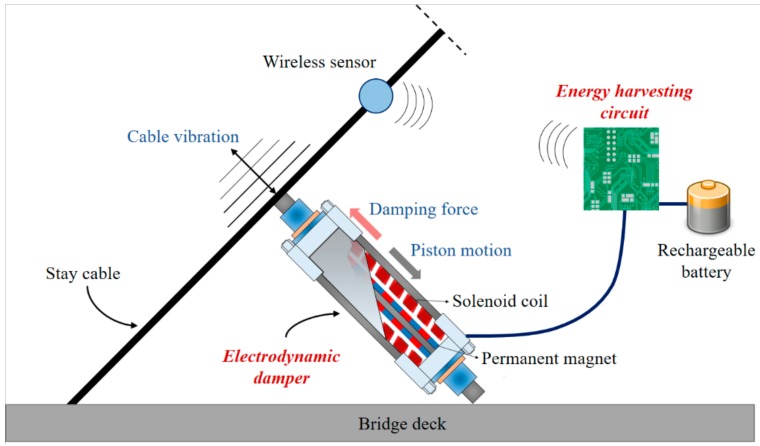
Outline of monitoring system using electrodynamic damper and energy harvesting circuit.

The design specifications of the electrodynamic damper determine the motor constant, damping force, and the electrical characteristics that appear in the circuit. Prior to the fabrication of the electrodynamic damper, major parameters that determine the damping performance were discussed. And to verify the energy harvesting performance, the energy harvesting circuit was designed and connected to the electrodynamic damper. The circuit was designed and manufactured considering the vibration response of the cable and the electrical output characteristics. The overall outline of the monitoring system using an electrodynamic damper and an energy harvesting circuit is shown in Figure 1.

### 2.1. Major Variables for Designing Electrodynamic Damper

An electrodynamic damper, which is a type of regenerative damping device, uses the electromagnetic force generated in the process of converting the mechanical vibration of a structure into electric energy as a vibration control force for the structure [29].

The electromagnetic force of an electrodynamic damper is proportional to the damper coefficient and speed, similar to that in a conventional damper, and can be expressed using Equation (1).

(1) $$F_{d}=c_{d}\cdot v\;.$$

In electrodynamic dampers composed of coils and magnets, the magnetic flux depending on the size and shape of the magnets and the time delay due to the inductance of the coils must be considered [30,31]. The relationship between the damping force and the speed considering the time delay due to the magnetic flux and inductance acting inside the cylindrical electrodynamic damper is as follows:

(2) $$L_{coil}\frac{dF_{d}}{dt}+R_{coil}\cdot F_{d}\;=\;K_{t}^{2}v\;,$$

where $L_{coil}$ is the inductance of the damper coil, $R_{coil}$ is the resistance of the damper coil, and $K_{t}$ is the machine constant of the damper. According to Equation (2), the relationship between damping force and speed, which governs the dynamic behavior of the damper, is determined by the machine constant.

Considering the linear estimation equation of the demagnetization curve, the gap magnetic flux density, and the pole shoes flux density at the interface of the cylindrical surface, the magnetic flux density equation can be derived as follows:

(3) $$B_{m}\;=\;\frac{B_{rm}\tau_{m}\tau_{f}}{\tau_{m}\tau_{f}+r_{m}^{2}\frac{\mu_{rp}}{\mu_{0}}\left(ln\left(\frac{r_{s}}{r_{m}}\right)+\frac{1}{2\mu_{Fe}}+\frac{\tau_{p}\tau_{f}}{\mu_{Fe}h_{y}\left(r_{s}+r_{e}\right)}\right)}\;.$$

where $B_{rm}$ is the remanence or residual flux density, $\mu_{rp}$ is the recoil permeability, $\mu_{0}$ is the free space permeability, $\mu_{Fe}$ is the relative permeability of iron. Other variables of the Equation (3) are shown in Table 1, which show the major design parameters of a cylindrical linear-type electrodynamic damper [32]. The total open circuit voltage induced from the damper by the series turns is

(4) $$V_{emf}\;=\;\pi pN_{a}B_{m}\frac{r_{m}^{2}}{\tau_{f}}v=K_{t}v\;,$$

where $K_{t}$ is an electromotive force constant of the machine. Therefore, the machine constant can be expressed by Equations (3) and (4) as follows:

(5) $$K_{t}\;=\;\frac{\pi pN_{a}r_{m}^{2}B_{rm}\tau_{m}}{\tau_{m}\tau_{f}+r_{m}^{2}\frac{\mu_{rc}}{\mu_{0}}\left(ln\left(\frac{r_{s}}{r_{m}}\right)+\frac{1}{2\mu_{Fe}}+\frac{\tau_{p}\tau_{f}}{\mu_{Fe}h_{y}\left(r_{s}+r_{e}\right)}\right)}\;.$$

According to Equation (5), the machine constant is expressed in terms of the geometric and magnetic properties of the device, and determines the force–velocity constitutive relation of the damper with the electrical circuit parameters.

The damping coefficient $c_{d}$ of the electrodynamic damper considering the time delay can be expressed using Equation (6):

(6) $$c_{d}\;=\;\frac{K_{t}^{2}}{\sqrt{{\left(R_{total}\right)}^{2}+{\left(\omega L_{coil}\right)}^{2}}}\;,$$

where ω is the natural frequency of the input. The inductance is affected by the excitation frequency due to the external load. When the external load is in the high-frequency range, the effect of the inductance is significant. However, as the natural frequencies of most civil structures are in the low-frequency region, the effect of the inductance on the damping coefficient of the proposed electrodynamic damper is negligible.

**Figure 2:**
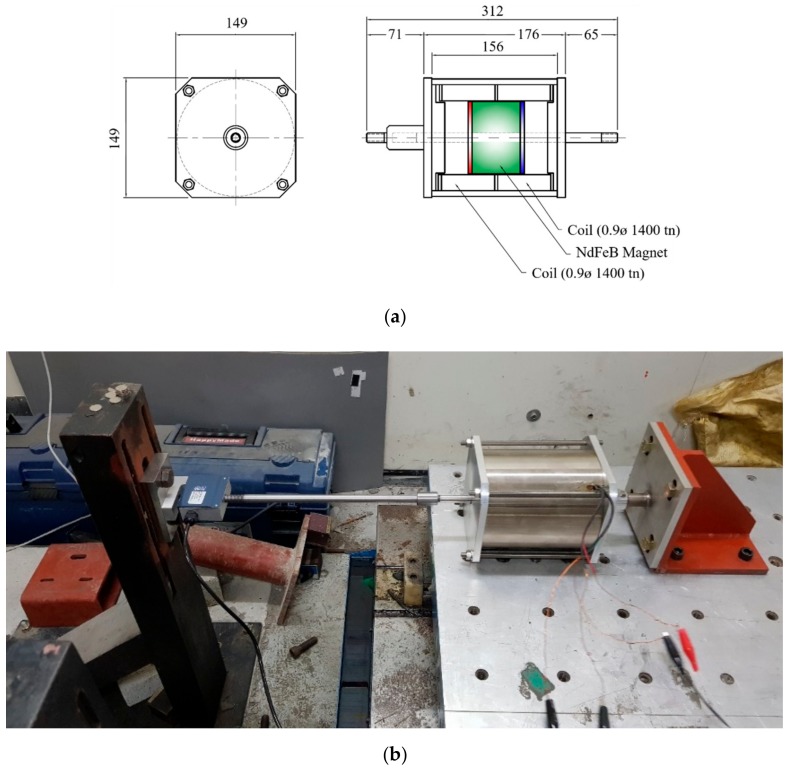
Production drawings and shaking table experiment view of the electrodynamic damper: (**a**) Drawing of an electrodynamic damper; (**b**) An electrodynamic damper mounted on a shaking table.

To experimentally verify the performance of the electrodynamic damper, an electrodynamic damper of the type shown in Figure 2 was fabricated. Table 2 lists the major design variables considered in the fabrication. Figure 2a shows a drawing of the manufactured electrodynamic damper. Figure 2b shows a damper mounted on a shaking table for the characteristic test. As the electrodynamic damper has two separate cylindrical solenoid coils wound inside it, the four wires were exposed to the outside of the damper and connected to an external circuit.

### 2.2. Energy Harvesting Circuit Configuration

An energy harvesting circuit was constructed to store and utilize the electric energy obtained from the vibration of the cable. It is difficult to attain a high voltage because the vibrations generated by civil engineering structures are relatively low compared with the vibrations generated by mechanical structures [33]. And because the electrodynamic damper generates alternating current (AC) power, it must be converted to direct current (DC) to charge the battery. Thus, a buck-boost converter that can be applied to a wide range of voltages with high conversion efficiency, and a high-efficiency chipset that minimizes internal losses were used.

**Figure 3:**
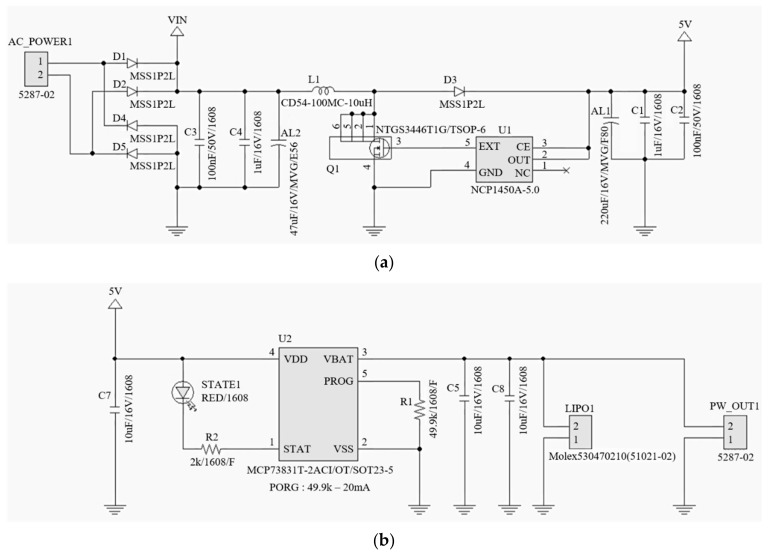
Energy harvesting circuit diagram: (**a**) Upper circuit diagram; (**b**) Lower circuit diagram.

Figure 3 is part of a circuit diagram built for energy acquisition. The upper circuit diagram consists of a diode bridge to convert AC generated from the damper to DC, a capacitor, an inductor and a transistor for managing power, and a DC-DC switching controller for supplying power to the battery pack. The switching controller implements a high efficiency converter. The lower circuit diagram consists of a charge controller and a connector for the lithium ion battery, and the circuit is composed of a typical application [34]. The circuit output was designed to output 4V or higher. The maximum current was allowed to flow at 20 mA and the voltage was kept constant for charging the battery.

## 3. Experimental Validation

### 3.1. Verification of Vibration Control Performance of Damper

To experimentally verify the vibration control performance of the electrodynamic damper, a cable of a footbridge in service was employed. The cable length was 11.8 m, the mass per unit length was 4.229 kg/m, and the diameter was 28.6 mm. The cable was insulated with a high-density polyethylene. Table 3 lists the other detailed specifications.

The electrodynamic damper was installed at 5% of the total cable length from the ground. An accelerometer and a laser displacement meter were installed at the position where the damper was installed. Accelerometers were installed at quarter, half, and three-fourth distances along the cable length to measure the response of the cable.

**Figure 4:**
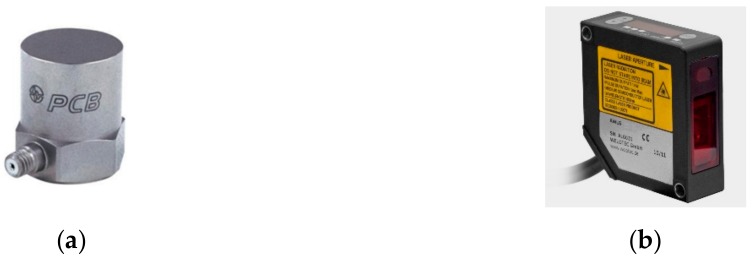
Measuring instruments: (**a**) Accelerometer; (**b**) laser displacement meter.

The accelerometer used was a 353B33 model from PCB Piezotronics, Inc. (Depew, NY, USA). The accelerometer is a single-axis model, which is mounted on a magnetic base to match the vibration direction due to the cable exciter. The laser displacement meter used was an AWLG 120S model from WeloTec Corp. (Laer, Germany). The appearance of each measuring instrument is shown in Figure 4. The specifications of each measuring instrument are shown in Table 4.

**Figure 5:**
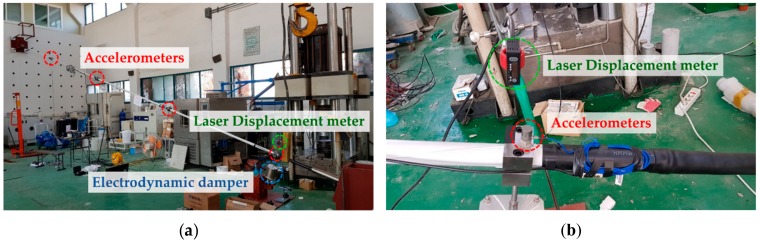
Experimental view: (**a**) Overall view; (**b**) details of measurement equipment around damper.

Figure 5 shows the cable structure, electrodynamic damper, and measuring instruments. Experiments on the cable structure were carried out separately by conducting a free vibration test and a forced vibration test. In the free vibration test, the damping ratio of the passive electrodynamic damper was evaluated by Hilbert transform. The variation in the damping ratio according to the magnitude of the external resistance was analyzed in the same manner as in a previous hybrid simulation [35].

Hilbert transforms are used to derive analytic signals from the phase shift of the signal and are often used to estimate the damping ratio. The free attenuation response in the 1st mode of the cable can be assumed to be a Hilbert transform and can be expressed as:

(7) $$U\left(t\right)=\;e^{-\xi \omega t}\cos\left(\omega_{d}t+\phi \right)\;,$$

where ξ is a modal damping ratio, ω is a modal frequency, and $\omega_{d}$ is a damped modal frequency. The Hilbert transform of the time domain value $U\left(t\right)$ can be expressed as:

(8) $$V\left(t\right)\;=\;-\frac{1}{\pi }\int_{-\infty }^{\infty }\frac{U\left(\eta \right)}{\eta -t}d\eta \;=\;e^{-\xi \omega t}\cos\left(\omega_{d}t+\phi \right)\;.$$

Thus, the analytical signal can be expressed as:

(9) $$H\left(t\right)\;=\;e^{-\xi \omega t}\cos\left(\omega_{d}t+\phi \right)+je^{-\xi \omega t}\sin\left(\omega_{d}t+\phi \right)\;=\;e^{-\xi \omega t}e^{j\left(\omega_{d}t+\phi \right)}\;,$$

(10) $$\left|H\left(t\right)\right|\;=\;e^{-\xi \omega t}\;,\;\;ln\left(\left|H\left(t\right)\right|\right)=\;-\xi \omega t\;,$$

where −ξω can be approximated from the slope of the straight line in the t-plane corresponding to $\left|H\left(t\right)\right|$. From the approximated straight line, the damping ratio corresponding to the modal frequency is calculated as:

(11) $$\omega \;=\;\sqrt{\omega_{d}^{2}+{\left(\xi \omega \right)}^{2}}\;,\;\xi \;=\sqrt{1-{\left(\frac{\omega_{d}}{\omega }\right)}^{2}}\;.$$

In the case of the forced vibration test, an exciter for the cable was produced separately, and the cable was excited at its natural frequency. The experiments were carried out using an unbalanced mass exciter, which was fabricated separately for cable excitation, to impart vibration at the natural frequency of the cable. The time and frequency-domain responses were compared. Table 5 lists the experimental cases to confirm the vibration control performance of the electrodynamic damper.

#### 3.1.1. Free Vibration Test

**Figure 6:**
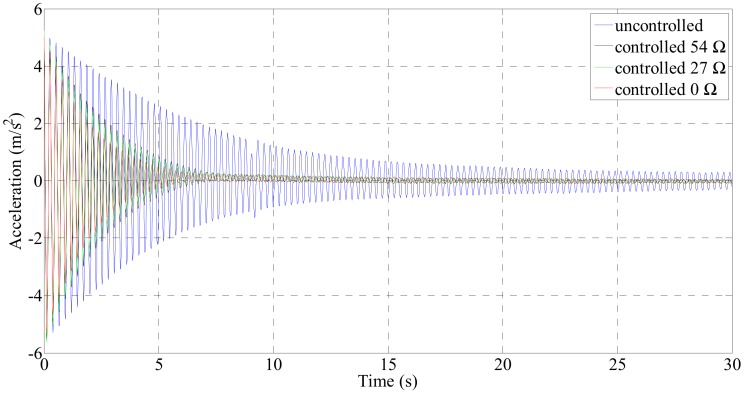
Acceleration response at cable center in free vibration test.

Figure 6 shows the acceleration response at the center of the cable structure in the free vibration test. To analyze the change in the damping ratio of the passive electrodynamic damper, the center of the cable was excited at the first natural frequency, the acceleration response at the center was measured, and the damping ratio change according to the external resistance change was examined. The change in the damping ratio according to the acceleration response was examined using the Hilbert transform.

**Figure 7:**
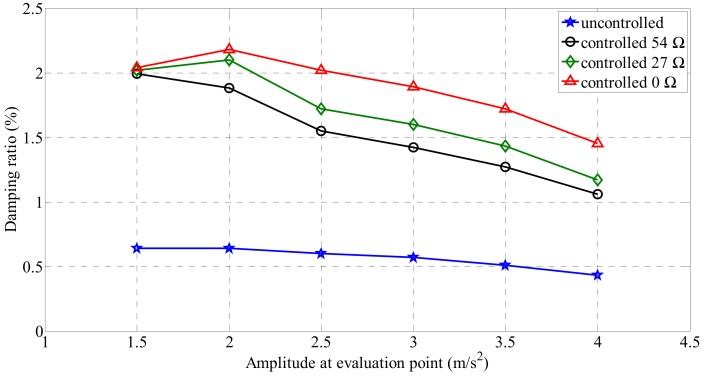
Damping ratio changes according to acceleration response.

Figure 7 shows the change in the damping ratio with the magnitude of the acceleration response using the Hilbert transform. An uncontrolled state means that no damper is attached to the cable, and the controlled 0 Ω state means that the damper is mounted on the cable and the coils are connected in series. In this case, the resistance of the circuit is 27 Ω, which is the resistance of the electrodynamic damper itself. The maximum damping ratio of the cable in the uncontrolled state was 0.64%. As the cable response decreased, the damping ratio increased overall. The average damping ratio at each evaluation point was 0.57%. Compared with the uncontrolled case, the passive electrodynamic damper improves the damping ratio of the cable.

The change in the damping ratio according to the magnitude of the external resistance when the electrodynamic damper is passively controlled was analyzed. The results showed that the damping ratio of the cable at all amplitude evaluation points decreased with the increase in the external resistance. The cable damping ratio according to the magnitude of the external resistance exhibited a constant change with the increase in the magnitude of the acceleration response. On the other hand, with the decrease in the magnitude of the acceleration response, the damping ratio remains constant regardless of the magnitude of the external resistance. For a passive electrodynamic damper of 0 Ω, the maximum damping ratio at which the attenuation is maximum was 2.18%. The average damping ratio at the evaluation point amplitudes was 1.88%. Table 6 lists the damping ratios and mean values for each acceleration response.

#### 3.1.2. Forced Vibration Test

**Figure 8:**
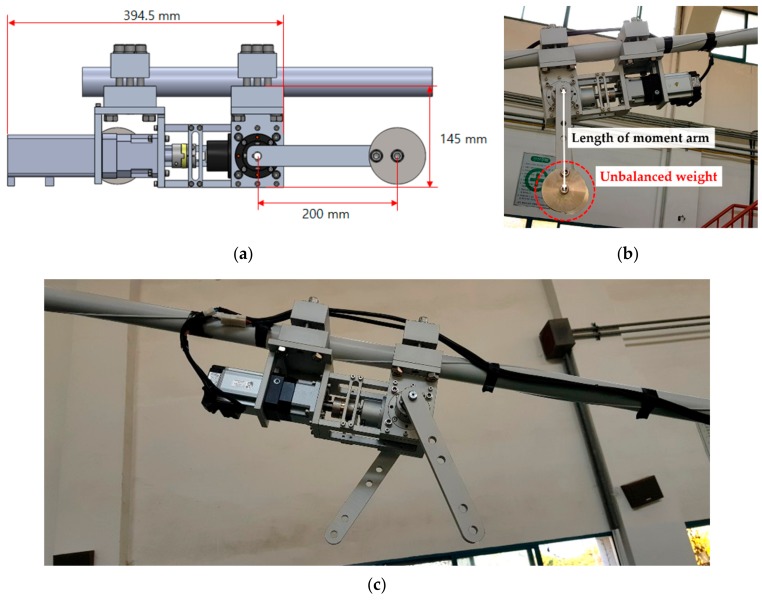
Cable exciter: (**a**) Drawing; (**b**) Exciter with unbalanced weight; (**c**) Exciter mounted on the cable without weight.

In the forced vibration test, the damping performance of the electrodynamic damper applied to the cable structure was evaluated using a specially designed exciter. The responses of the cable structure before and after damper installation were compared by applying the passive electrodynamic damper with an external resistance of 0 Ω, in which case the vibration damping performance in the free vibration test was the best. The mass weights attached to the cable exciter rotate in opposite directions. The excitation force acting perpendicular to the cable is generated by the unbalanced excitation. The force exerted on the cable was calculated using the following equations:

(12) $$F_{0}=\bar{m}e{\bar{\omega }}^{2}\;\mathrm{and}\;F\left(t\right)=\;F_{0}\sin\bar{\omega }t\;,$$

where $\bar{m}$ is the unbalanced mass of the cable exciter, *e* is the moment arm length of the cable exciter, and $\bar{\omega }$ is the angular velocity of the cable exciter. The torque of the cable exciter was set to be approximately 4 N·m. The frequency domain of the exciter is designed to maintain a constant torque up to a frequency of 10 Hz. Considering the acceleration torque and a safety factor of 1.5 for the exciter, the momentary maximum torque of the exciter is designed to reach up to 7.5 N·m. Figure 8 shows the design of the cable exciter and its installation position on the cable.

**Figure 9:**
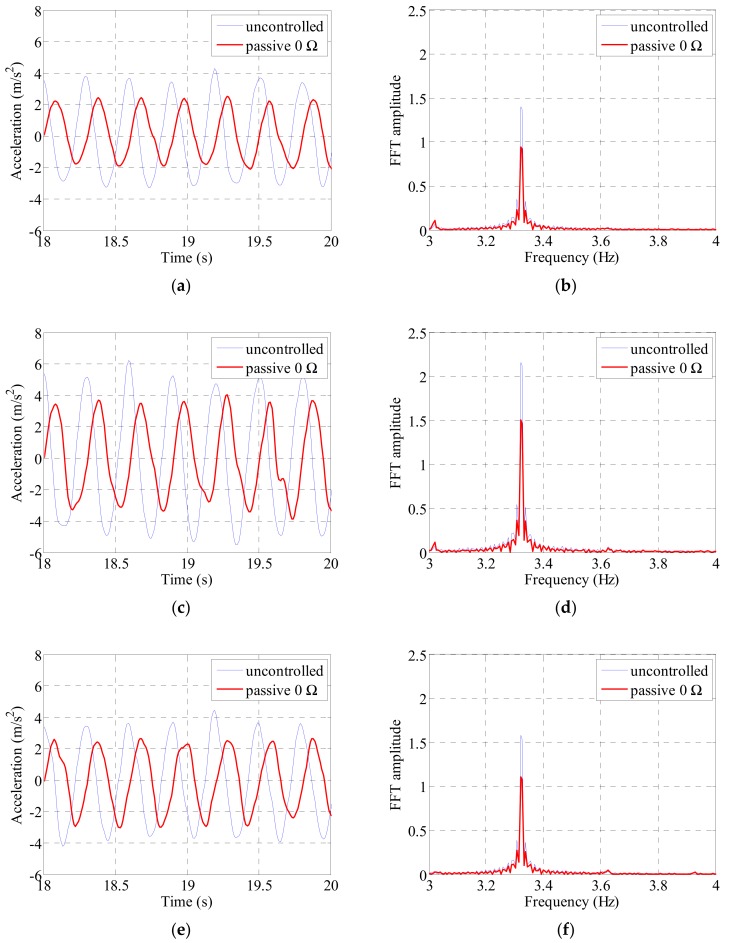
Cable acceleration and FFT (Fast Fourier Transform) response by sinusoidal excitation of 1st natural frequency: (**a**) 1/4 point acceleration; (**b**) 1/4 point FFT; (**c**) 1/2 point acceleration; (**d**) 1/2 point FFT; (**e**) 3/4 point acceleration; (**f**) 3/4 point FFT.

**Figure 10:**
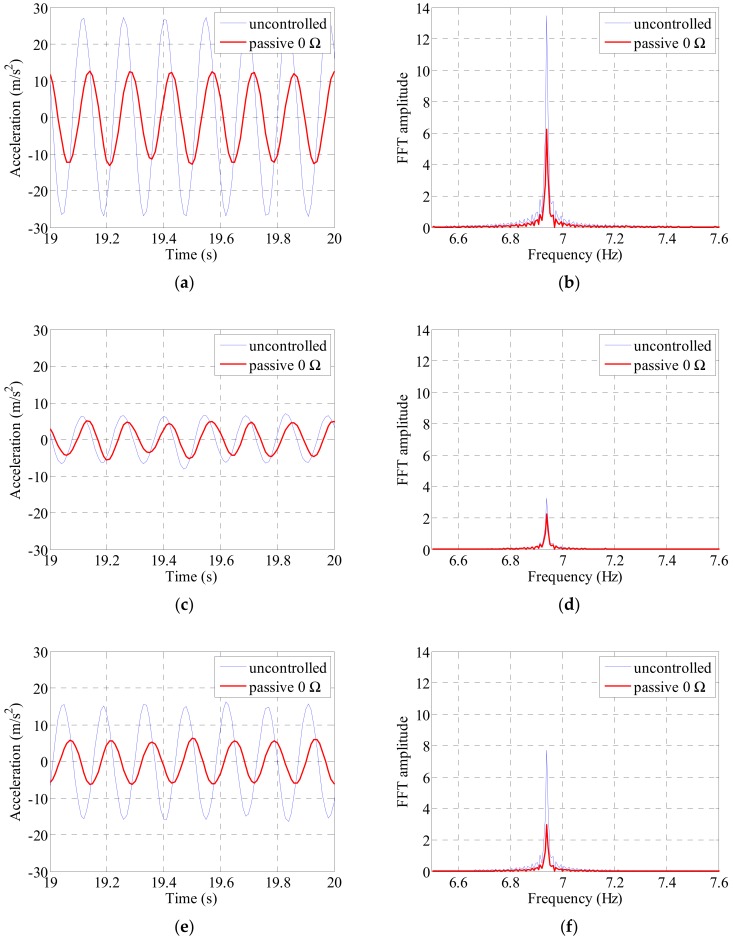
Cable acceleration and FFT response by sinusoidal excitation of 2nd natural frequency: (**a**) 1/4 point acceleration; (**b**) 1/4 point FFT; (**c**) 1/2 point acceleration; (**d**) 1/2 point FFT; (**e**) 3/4 point acceleration; (**f**) 3/4 point FFT.

**Figure 11:**
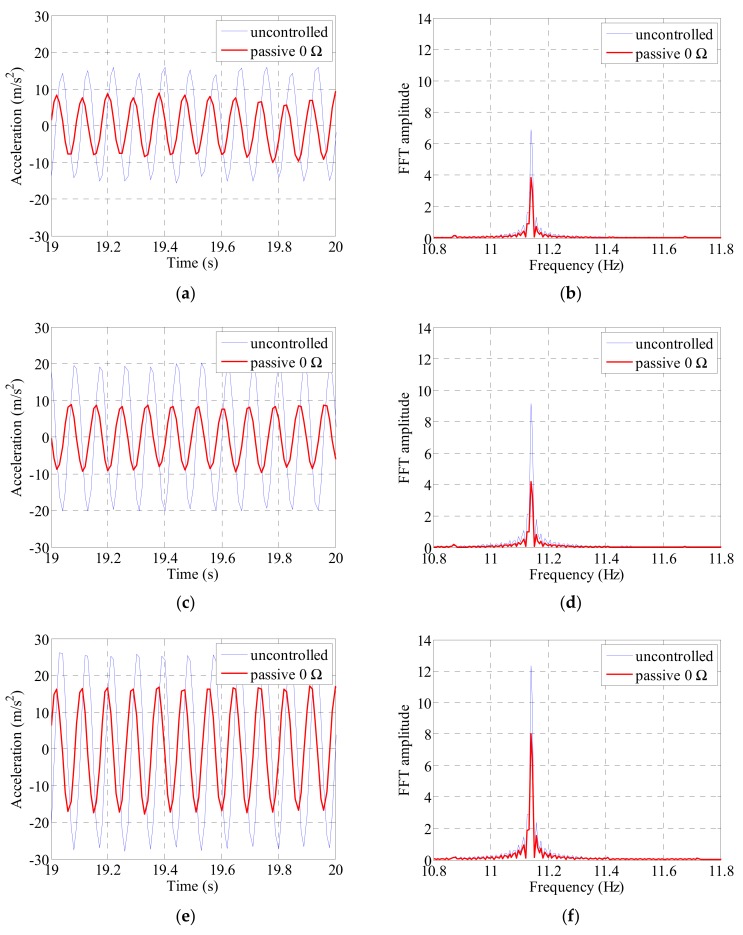
Cable acceleration and FFT response by sinusoidal excitation of 3rd natural frequency: (**a**) 1/4 point acceleration; (**b**) 1/4 point FFT; (**c**) 1/2 point acceleration; (**d**) 1/2 point FFT; (**e**) 3/4 point acceleration; (**f**) 3/4 point FFT.

**Figure 12:**
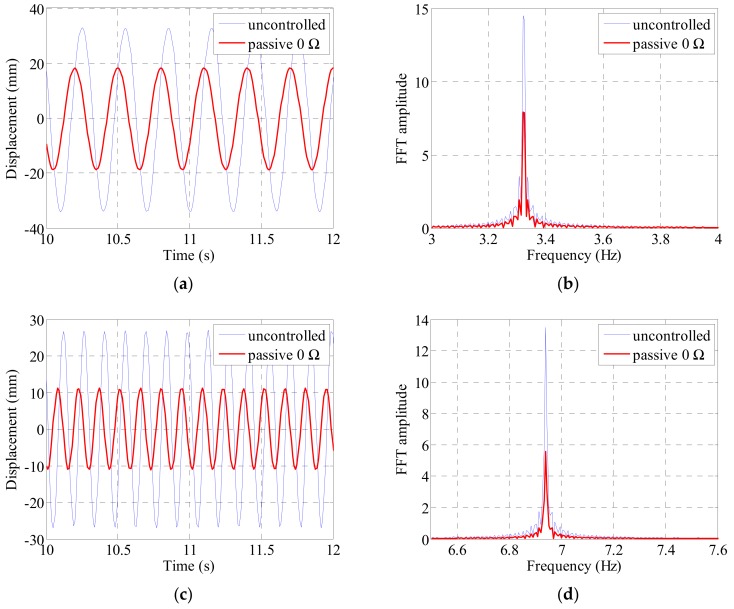
Cable displacement and FFT response by sinusoidal excitation at the 1st and 2nd natural frequency: (**a**) 1/2 point displacement at the 1st natural frequency; (**b**) 1/2 point FFT at the 1st natural frequency; (**c**) 1/4 point displacement at the 2nd natural frequency; (**d**) 1/4 point FFT at the 2nd natural frequency.

The cable was excited at the first to third natural frequencies of the cable. We compared the acceleration and cable center displacements before and after the electrodynamic damper installation and compared the magnitudes of the frequency-domain responses. The acceleration was measured at the 1/4, 1/2, and 3/4 points along the cable length and the displacement at the 1/4 and 1/2 points. Figure 9, Figure 10, Figure 11 and Figure 12 show the magnitude of the acceleration response, the displacement response, and the response magnitude in the frequency domain at each measurement point of the cable under natural frequency conditions.

As a result of examining in the time and frequency domain through the figures, it was confirmed that there is a distinct response difference according to the location of the cable in each natural frequency condition by the mode shape. Figure 9 is a graph showing the acceleration and the Fast Fourier Transform (FFT) response at each location of the cable under the condition of the first natural frequency excitation. The largest response size was found at half of the cable and a similar level of response reduction was seen at the 1/4 and 3/4 points. It can be seen that the response is reduced constantly at each point of the cable by mounting the electrodynamic damper. The FFT response size was the largest at 3.32 Hz, the first natural frequency of the cable, and a similar level of FFT response reduction was observed at other locations of the cable.

Figure 10 shows the acceleration and FFT response at each location of the cable under conditions with a second natural frequency excitation. The magnitude of the acceleration response in the time domain was largest at one quarter of the cable and smallest at half of the cable. Even if considering the node movement due to the mounting of the electrodynamic damper, the half-point of the cable is located close to the node when estimating the secondary mode shape of the cable, and therefore the damping effect by the electrodynamic damper is relatively small. The singularity is that the maximum value of the acceleration and the FFT response at the 1/4 point is about twice as large as that at the 3/4 point. This is because the mass distribution per unit length of the cable has changed irregularly due to the added mass of the exciter installed at the upper part of the cable and a slight deflection has occurred. The second natural frequency was 6.94 Hz, and the largest acceleration and FFT response reduction of the cable are observed at 3/4 position.

The acceleration and FFT response of each location of the cable under conditions with a third natural frequency excitation can be seen in Figure 11. The acceleration and FFT response magnitudes by excitation were the largest at 3/4 of the cable. And the largest FFT response was at the third natural frequency of the cable, 11.14 Hz. However, the greatest acceleration and FFT response reduction appeared at half the cable location. Similar to the experimental results with the second natural frequency, the asymmetric response based on the nodal point of the cable was shown in the third order. Acceleration and FFT maximum response at point 3/4 were about twice as large as at point 1/4, as in experiments with second mode, which were found to be due to asymmetrical mode shape in cable due to the additional mass of the cable exciter.

The acceleration and frequency-domain responses at each measurement point of the cable decreased when the passive electrodynamic damper was installed. Table 7 and Table 8 list the results. In the case of the first natural frequency, the acceleration and frequency-domain response reduced to approximately 70%. The vibration damping performance was found to be more pronounced when the vibration was excited at the second and third natural frequencies than that when the vibration was excited at the first natural frequency. The overall vibration reduction performance when excited at the second and third natural frequencies was 50% or more at the position where the main response of the cable appeared. The vibration reduction performance was relatively low at the center of the cable when excited at the second natural frequency. The overall damping performance decreased because the magnitude of the response at the center was relatively low when excited at the second natural frequency.

The RMS displacement of the cable was found to be reduced by 45% at the center of the cable when excited at the first natural frequency and by 49% at the quarter point when excited at the second natural frequency. As shown in Figure 12, a similar level of response reduction is observed in the frequency domain.

As shown in the characteristic test, the damping performance of the electrodynamic damper gradually increased with the frequency of the external load and is maximum at natural frequencies of 5 Hz or more. As the first natural frequency of the cable used in the experiment was 3.32 Hz, the damping performance of the damper was not maximum. As a result, the damping performances at the second and third natural frequencies are relatively excellent. Generally, the main response of a cable in a bridge is in the secondary and tertiary natural frequency ranges. Therefore, it is considered that the relatively good damping performance of the electrodynamic damper at the second and third natural frequencies is sufficient for the vibration control of the cable.

### 3.2. Energy Harvesting Verification Based on Field Measurement Data of Bridge in Service

To evaluate the energy harvesting performance of the electrodynamic damper, the power production performance was evaluated using field measurement data. We used the acceleration data of a cable measured at the second Jindo Bridge as the field measurement data.

**Figure 13:**
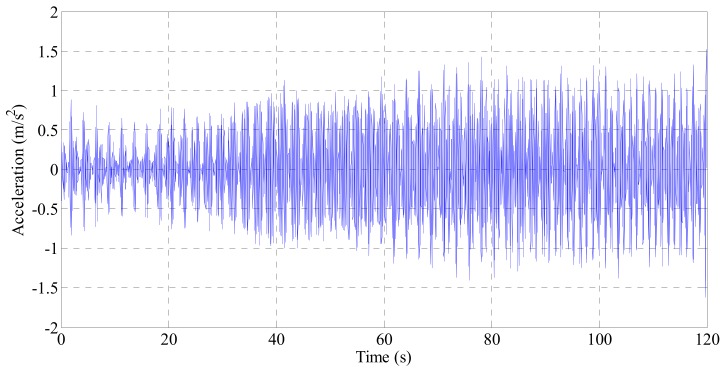
Acceleration response value of cable by field measurement.

The acceleration data measured for the cable of the second Jindo bridge is shown in Figure 13. The RMS acceleration of the cable was 0.73 m/s^2^, and the mean wind speed was 5.4 m/s. The measured acceleration data were converted into cable displacement and used as the excitation source data for the electrodynamic damper. The electrodynamic damper was excited using a shaking table, and the power obtained through the energy harvesting circuit was measured.

**Figure 14:**
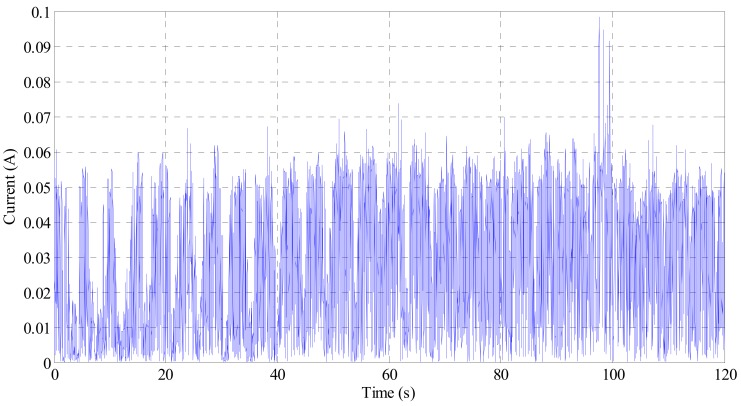
Current output from the electrodynamic damper.

Figure 14 shows the current obtained from the electrodynamic damper at an average wind speed of 5.4 m/s [36]. The energy harvesting circuit connected to the electrodynamic damper produces 5 V, direct current. At a wind speed of 5.4 m/s, the power output of the electrodynamic damper was approximately 174.6 mW. To operate the wireless sensor, it is necessary to consider the actual power produced from the electrodynamic damper, the power consumed by the wireless sensor, and the power loss of the battery itself [37].

Table 9 lists the power consumptions of Imote2, an advanced wireless sensor node platform, for each operation. According to the table, Imote2 consumes 26.14 mWh once a day and 41.48 mWh when measured twice a day. When the average wind speed was 5 m/s, the power output of the electrodynamic damper obtained through the hybrid simulation was sufficient to operate the wireless sensor.

The power generated from the acceleration data of the cable on the second Jindo Bridge was 174.6 mW, enough to operate the Imote2 wireless sensor. The power loss of the battery itself was 10%. Although the power loss of the battery itself is taken into consideration, it has been confirmed that the power produced by the electrodynamic damper is sufficient to operate the wireless sensor.

## 4. Operation Strategy of Monitoring System Equipped with Electrodynamic Damper

To apply the electrodynamic damper with a vibration control and energy harvesting function to the cable of the bridge, a proper damper operation plan is needed. A continuous energy harvesting performance is required to operate a wireless sensor for cable monitoring. To operate the wireless sensor, more energy is required considering the energy loss of the battery itself [39].

**Figure 15:**
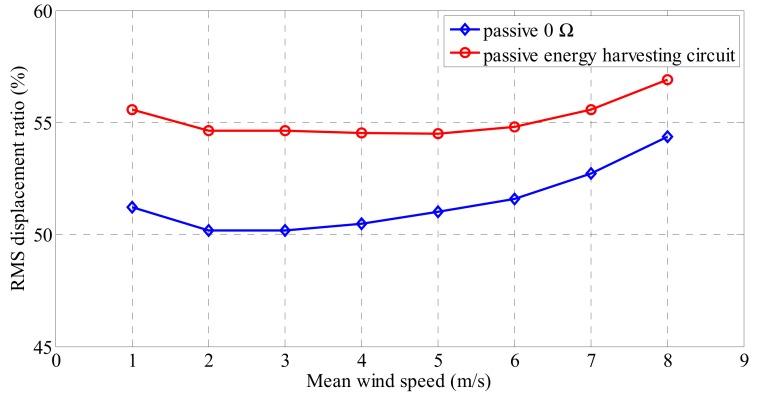
RMS displacement ratio according to mean wind speed.

The wind loads acting on the cables of the bridge vary continuously. Therefore, the performance of the electrodynamic damper needs to be controlled according to the change in the wind speed. A charging circuit is required to store the electrical energy produced by the electrodynamic damper. Connecting the charging circuit to the electrodynamic damper causes additional circuit resistance, which reduces the overall vibration control performance of the damper. Figure 15 shows the relative reduction rate of the RMS displacement in terms of the mean wind speed due to energy harvesting circuit connections. The damping performance decreases under all wind speed conditions. As the vibration control of the electrodynamic damper is not required when the wind speed is low, the proposed damper can be utilized as an energy source for the wireless sensor for monitoring. However, when the cable vibration is excessive because of high wind speed, a proper vibration control method is required.

To propose an operation plan for the electrodynamic damper, it is necessary to analyze the wind environment around the bridge. The distribution of the wind velocity acting on the bridge should be analyzed, and the electrical energy obtained from the electrodynamic damper should be calculated. Finally, considering the power consumption of the wireless sensor and the power loss of the battery, the operation plan of the electrodynamic damper should be proposed.

**Figure 16:**
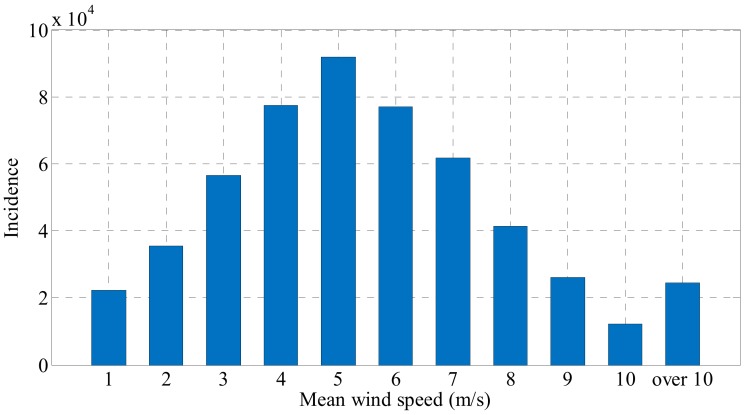
One-minute mean wind speed distribution in Jindo (2016).

According to the meteorological agency, the average annual wind speed observed at the meteorological station around the second Jindo Bridge was 4.76 m/s. Figure 16 and Table 10 present the one-minute mean wind speed observed at the meteorological center around the second Jindo Bridge in 2016.

Based on the one-minute mean wind speed distribution of the second Jindo bridge, the electric power production of the electrodynamic damper can be predicted. The power produced by the electrodynamic damper was calculated through the hybrid simulation [40]. The power output of the electrodynamic damper was estimated based on the ratio of the one-minute average wind speed under each wind speed condition and the power output of the electrodynamic damper at the corresponding wind speed.

As a result of applying the power output of the electrodynamic damper at an average wind speed in the range of 1–8 m/s and the distribution ratio of the corresponding wind speed, the power output of the electrodynamic damper was estimated to be approximately 159.5 mWh. When performing data measurement using Imote2, the power required for n measurements is 10.8 + 15.34 × *n* mWh. The power consumption of general lithium ion batteries itself is approximately in the range of 5–10%. Assuming that the power efficiency of the lithium ion battery is 90%, the possible amount of power through the electrodynamic damper was estimated to be approximately 143.55 mWh. This is enough energy for the wireless sensor to perform eight measurements a day. However, one of the biggest disadvantages of lithium ion batteries is stability, which is greatly affected by temperature. The annual temperature in the area of the second Jindo bridge over the past year has ranged from –2 to 29 degrees Celsius. Later on, in order to actually build a monitoring system on the site, the stability of the battery should be reviewed according to the temperature condition of the area.

**Figure 17:**
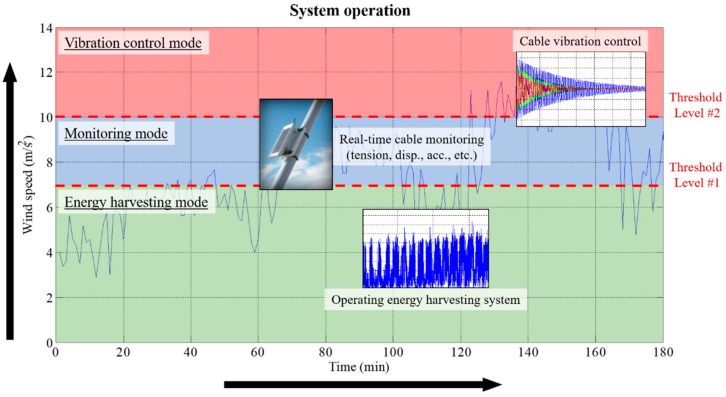
Overview of electrodynamic damper operation strategy.

The operating steps of the electrodynamic damper for the cable structure are divided into three stages. Figure 17 shows the operating strategy of the electrodynamic damper according to the wind speed.

The first stage is the energy harvesting stage, in which the electrodynamic damper is operated for energy harvesting. The energy harvesting stage is when the wind speed on the cable is in the range of 1–6 m/s, which is 68.4% of the total wind velocity distribution on the second Jindo Bridge. At this stage, the variable resistance value connected to the damper is at its maximum. The variable resistance value changes in a direction that minimizes the damping performance and maximizes the energy harvesting performance. Energy is continuously harvested from the electrodynamic damper, and the wireless sensor operates twice a day.

The second stage is the monitoring stage, wherein the wind speed on the cable is in the range of 7–10 m/s. In the monitoring stage, the energy harvesting of the electrodynamic damper is performed continuously; however, the measurement frequency of the wireless sensor increases from 2 to 8 times. As mentioned earlier, the power available through the electrodynamic damper is the amount of energy that the wireless sensor can measure by operating more than eight times a day for the Imote2 wireless sensor. It is possible to perform the operation such that the monitoring frequency of the wireless sensor is increased with the increase in the speed of the wind acting on the cable. At this stage, the variable resistance value connected to the electrodynamic damper has an intermediate value. The damper performs both damping and energy harvesting functions at the same time, and is prepared to cope with larger vibration. In addition, the number of measurements in the monitoring stage is expected to increase dramatically when various low power wireless sensors under development are applied.

The final operating stage of the electrodynamic damper is the vibration control stage. This is when the speed of the wind acting on the cable is 10 m/s or higher. In the vibration control stage, the electrodynamic damper operates in a direction to improve the vibration control performance. In the vibration control stage, the energy harvesting circuit mounted on the electrodynamic damper is switched to vibration control. If a wind speed of 10 m/s or higher causes excessive vibration of the cable, the electrodynamic damper is separated from the energy harvesting circuit, and the damping performance of the damper is maximum. To this end, the external resistance value is minimized and only the damper itself acts as a resistor. The wireless sensor is monitored continuously, even in the vibration control stage wherein no energy harvesting is performed. Because the power of the wireless sensor is supplied through the battery, continuous energy supply is possible without additional energy harvesting. As listed in Table 10, the rate of one-minute mean wind speed at which a high wind speed above 10 m/s occurs is approximately 4.7% per year. As continuously high wind speeds are rarely sustained, the electrodynamic damper is expected to operate in the vibration control stage only for a short duration. This means that the energy stored in the battery can be used to immediately activate the electrodynamic damper in the vibration control mode. Table 11 lists the overall operating strategy of the electrodynamic damper at various wind speeds.

## 5. Conclusions

To experimentally confirm the vibration control performance realized by mounting an electrodynamic damper, designed based on the major design variables, on a cable, the cable response and damping ratio under different excitation conditions of free vibration and natural frequency was examined. The results showed that the damping ratio decreased with the increase in the external resistance under all acceleration response conditions and free vibration condition and was constant regardless of the external resistance with the decrease in the acceleration response magnitude. For a passive electrodynamic damper with an external resistance of 0 Ω, the maximum damping ratio was 2.18%, and the average damping ratio at acceleration response evaluation points was 1.88%.

In the forced vibration test, the unbalanced mass rotating vibrator designed for the cable excitation was used to excite the cable at the 1st to 3rd natural frequencies, and the acceleration, displacement, and frequency-domain responses of each measurement point were examined.

The reduction in the acceleration response at the 1st to 3rd natural frequencies was up to 31.98%, 61.53%, and 50.04%, respectively, and the reduction in the frequency-domain response was up to 32.86%, 61.76%, and 54.05%, respectively. Under the second and third natural frequency excitation conditions, the response variation according to the measurement point was significant because of the mode shape. The RMS displacement of the cable was reduced by 45% at the center of the cable at the first natural frequency, by 49% at one quarter of the cable at the second natural frequency, and a similar level of response reduction was observed in the frequency domain. Considering the main response frequency of the cable of a general bridge, the proposed electrodynamic damper is expected to be effective for the vibration control of the cable.

To evaluate the energy harvesting performance, the field measurement acceleration data of an actual cable of a bridge in service were employed. The power output of the electrodynamic damper was approximately 174.6 mW at a mean wind speed of 5.4 m/s, sufficient to operate the wireless sensor of Imote2 even considering the consumed power, measurement frequency, and battery loss of the wireless sensor.

The meteorological data of the area where the bridge in service was located were analyzed, and the power output according to the wind speed condition considering the energy harvesting performance of the electrodynamic damper was calculated. The results showed that the damper can be used in a cable vibration monitoring system by dividing its operation into three stages depending on the wind environment: energy harvesting, monitoring, and vibration control.

Experimental and analytical studies carried out in this study are expected to be useful for developing an electrodynamic damper and energy harvesting circuit to reduce cable vibration and to build a monitoring system. The electrodynamic damper proposed in this study can be improved by applying the eddy currents, Halbach arrangement, etc. And it can be expected that the damping performance suitable for the purpose can be shown by the optimization through the electromagnetic analysis. By constructing the monitoring system and applying it to the cable of bridge in the field, I think that we can find more various complementary points that were not found at the level of current research. Future research will be carried out on improving the performance of the damper by developing and optimizing the hybrid electrodynamic damper through electromagnetic analysis.

## Figures and Tables

**Table 1:** Design parameters of electrodynamic damper

Parameters	Symbol	Explanation
Pole pitch	$\tau_{p}$	Distance between polarity changes
Magnet length	$\tau_{m}$	Actual length of the magnet
Pole shoe width	$\tau_{f}$	Width of the pole shoes
Air gap	g	Distance between the mover and the armature windings
Number of poles	p	Even number of poles in the machine
Coil height	$h_{w}$	Height of the coils in the armature
Coil width	$\tau_{w}$	Width of each coil in the armature
Wire radius	$r_{w}$	Radius of the coil wire
Coil turns	$N_{w}$	Number of turns on each coil
Active coil turns	$N_{a}$	Number of turns on each coil intercepted by the pole shoe flux
Mover radius	$r_{m}$	Radius to the outside surface of the magnet
Armature radius	$r_{i}$	Radius to the inside surface of the armature
Stator yoke radius	$r_{s}$	Radius to the inside surface of the stator yoke
Machine radius	$r_{e}$	Radius to the outer surface of the machine
Yoke thickness	$h_{y}$	Thickness of the armature shell

**Table 2:** Major design variables and values of electrodynamic damper considered in the fabrication.

Parameter	Symbol	Value	Parameter	Symbol	**Value**
Pole pitch	$\tau_{p}$	0.07 m	Wire radius	$r_{w}$	0.0045 m
Magnet length	$\tau_{m}$	0.06 m	Coil turns	$N_{w}$	1400
Air gap	g	0.001 m	Coil resistance	R	27 Ω
Number of poles	p	1	Yoke thickness	$h_{y}$	0.02 m
Coil height	$h_{w}$	0.02 m			

**Table 3:** Specification of cable.

Properties	Value	Properties	Value
Mass per unit length	4.229 kg/m	Inclination	18.72°
Cable length	11.8 m	Cross-sectional area	0.0014 m^2^
Diameter	42.2 mm (with insulation)	Location of damper	5% of cable length from the ground
Cable tension	50 kN		

**Table 4:** Specification of measuring instruments.

Sensor	Specification	Value
Accelerometer	Sensitivity	(±5%) 100 mV/g (10.19 mV/(m/s^2^))
	Measurement Range	±50 g (±491 m/s^2^)
	Broadband Resolution	0.0005 g RMS ^1^ (0.005 m/s^2^ RMS)
	Frequency Range	(±5%) 1 to 4000 Hz
	Sensing Element	Quartz
	Weight	0.95 oz (27 gm)
Laser displacement meter	Measuring range	60–180 mm
	Resolution	8 µm
	Linearity error	0.12 mm
	Measuring frequency	5000 Hz
	Current output	3.2–20.8 mA
	Voltage output	0–10.5 V

^1^ RMS: root mean square.

**Table 5:** Excitation conditions for cable experiments.

Case	Description	Parameter
#1–3	Free vibration test	External resistance: 0, 27, and 54 Ω
#4–6	Forced vibration test	Sinusoidal input at the 1st, 2nd, and 3rd natural frequencies

**Table 6:** Damping ratio (%) corresponding to different acceleration responses and external circuit resistances.

Acceleration Response	1.5 m/s^2^	2.0 m/s^2^	2.5 m/s^2^	3.0 m/s^2^	3.5 m/s^2^	4.0 m/s^2^	Average
Uncontrolled	0.64	0.64	0.60	0.57	0.51	0.43	0.57
Controlled 0 Ω	2.04	2.18	2.02	1.89	1.72	1.45	1.88
Controlled 27 Ω	2.02	2.10	1.72	1.60	1.43	1.17	1.67
Controlled 54 Ω	1.99	1.88	1.55	1.42	1.27	1.06	1.53

**Table 7:** RMS acceleration response and reduction ratio.

		¼ L ^1^ acc. ^2^	½ L acc.	¾ L acc.
1st natural frequency	Uncontrolled	2.3111	3.5705	2.5970
	Controlled	1.5720 (68.02%)	2.4958 (69.90%)	1.8451 (71.05%)
2nd natural frequency	Uncontrolled	19.1510	4.6230	10.9900
	Controlled	8.8945 (46.44%)	3.2403 (70.09%)	4.2277 (38.47%)
3rd natural frequency	Uncontrolled	10.5704	14.0073	18.9068
	Controlled	5.9262 (56.06%)	6.4375 (49.96%)	12.2075 (64.57%)

^1^ L: total length of the cable, ^2^ acc.: acceleration (m/s^2^).

**Table 8:** FFT amplitude and reduction ratio of acceleration.

		¼ L ^1^ FFT amp. ^2^	½ L FFT amp.	¾ L FFT amp.
1st natural frequency	Uncontrolled	1.40	2.16	1.58
	Controlled	0.94 (67.14%)	1.50 (69.44%)	1.11 (70.25%)
2nd natural frequency	Uncontrolled	13.48	3.25	7.74
	Controlled	6.23 (46.22%)	2.23 (68.62%)	2.96 (38.24%)
3rd natural frequency	Uncontrolled	6.90	9.14	12.32
	Controlled	3.84 (55.65%)	4.20 (45.95%)	8.02 (65.08%)

^1^ L: total length of the cable, ^2^ amp.: acceleration amplitude (m/s^2^).

**Table 9:** Estimated daily power consumption of Imote2 [38].

Mode	Current (mA)	Power (mW)	Duration	Power Consumption (mWh)
Wake-up	48	216	30 s (× n times)	1.8 (× n times)
Sensing	169	760.5	49.6 s (× n times)	10.48 (× n times)
Data processing	80	360	9.6 s (× n times)	0.96 (× n times)
Data transfer	55	247.5	30.8 s (× n times)	2.12 (× n times)
Sleep	0.1	0.45	24 h – (120 s × n times)	10.8 – 0.015 × n times
Total				10.8 + 15.34 × n times

**Table 10:** One-minute mean wind speed distribution value in Jindo (2016).

Mean Wind Speed (m/s^2^)	1	2	3	4	5	6	7	8	9	10	Over 10
Incidence ratio (%)	4.2	6.8	10.7	14.7	17.4	14.6	11.7	7.8	5.0	2.3	4.7

**Table 11:** Operation plan of electrodynamic damper

Wind Speed (m/s)	Electrodynamic Damper Operation	Measurement Count
0–6	Energy harvesting stage (Electrodynamic damper operates as an energy harvesting device)	2 times per day
7–10	Monitoring stage (Electrodynamic damper operates as an energy harvesting device and simultaneously increases the measurement frequency of the wireless sensor)	8 times per day (maximum count)
Over 10	Vibration control stage (Electrodynamic damper operates as a vibration control device and maximizes the vibration control performance)	8 times per day (maximum count)
